# Control Efficacy of UAV-Based Ultra-Low-Volume Application of Pesticide in Chestnut Orchards

**DOI:** 10.3390/plants12142597

**Published:** 2023-07-09

**Authors:** Takumi Arakawa, Shinji Kamio

**Affiliations:** Gifu Prefectural Research Institute for Agricultural Technology in Hilly and Mountainous Areas, Nakatsugawa 508-0203, Japan; kamio-shinji@pref.gifu.lg.jp

**Keywords:** *Castanea* ssp., pest control, precision agriculture, droplet deposition

## Abstract

Pesticide spraying using unmanned aerial vehicles (UAVs) has been utilized in many crops, including fruit tree crops, because of its merits in terms of labor-saving and the low risk to the operator. However, its relevance to chestnut, one of the commercially significant fruit trees grown throughout Europe and Asia, has not been studied. In this work, we assessed the effectiveness of UAV-based ultra-low-volume pesticide application in chestnuts. We demonstrated the efficiency of three insecticides applied by a UAV on young chestnut trees. Interestingly, using a reduced amount of one of the pesticides, UAV-based spraying had greater control efficacy than conventional methods. The efficacy of ultra-low-volume pesticide application to adult trees was equivalent to using an air-blast sprayer. The spray coverage was compared in terms of spray volume (20 L vs. 40 L ha^−1^), flight method (straight flight vs. rotating flight for each tree), the size of the UAVs (8 L vs. 30 L in payload capacity), flow rate (3.8 L vs. 6.0 L min^−1^), and tree age in order to characterize the droplet deposition of UAV-based spraying. Overall, we showed that spraying pesticides using a UAV could effectively protect chestnut trees. It was debated how tree training, or tree height, affected pest control.

## 1. Introduction

Chestnuts (*Castanea* sp.), which are edible nuts with nutritional benefits (such as low fat and high vitamin C), are vital [1]. In total, 2.3 million metric tons of chestnut fruits are produced worldwide across 568,175 hectares of chestnut orchards, and they are mostly grown in Europe and Asia [2]. The gall wasp (*Dryocosmus kuriphilus* Yasumatsu), Lepidoptera species (*Cydia* sp.), and weevil (*Curculio* sp.) are the major insect pests in chestnut as problems for fruit production [1]. Gall wasp caused serious damage to chestnut trees during the 1950s in Japan [3], one of the major producers of chestnuts in Asia. The prevalence of the parasitoid, *Torymus sinensis* Kamijo and the development of tolerant cultivars led to a significant decline in gall wasp infestation rates around 1990 [4,5]. The chestnut weevil (*Curculio sikkimensis* Heller) and peach moth (*Conogethes punctiferalis* Guenée) are now known to be major pests in Japan and frequently cause yield loss by invading chestnut fruit with young larvae. As a result, pesticides have been used in many production areas through the use of ground sprayers, including high-pressure sprayers and air-blast sprayers, to protect against pest attack [6]. However, ground spraying operators run a significant risk of health problems, and fewer and older growers make it difficult to manage these pests effectively. Thus, an efficient application technique must be developed to boost or sustain chestnut production.

In Japan in the early 1990s, unmanned helicopters were equipped to spray ultra-low volumes of phenthoate (*S*-α-ethoxycarbonylbenzyl *O*,*O*-dimethyl-phosphorodithioate) [7]. However, this method was rarely used, possibly due to the fact that the production sites were located in mountainous areas and the tree heights were high, meaning that these areas were not suitable for unmanned helicopter flight. According to Lee et al. [8], aerial pesticide applications using unmanned aerial vehicles (UAVs) against pests would be somewhat effective, but the dilution rate of the pesticide would be the same as conventional ones, which was impractical due to the large required spray volume (>2000 L ha^−1^) and the UAVs’ 40 L maximum payload capacity. Therefore, to the best of our knowledge, no labor-saving, low-risk application approach has been developed for chestnut.

The application of UAVs in fruit tree management can achieve high-throughput and precise monitoring of tree growth and disease onset based on visible and/or near-infrared reflectance [9]. It has been used in chestnuts to estimate yield [10] and biomass [11] and to monitor tree health against major diseases, such as chestnut blight and ink disease [12,13].

UAV-based aerial agricultural pest management has gained popularity recently around the world [14]. This was also true for fruit and nuts trees, such as citrus, peaches, grapes, pears, olives, apples, almonds, and papaya [15,16,17,18,19,20,21,22]. In comparison to unmanned helicopters or ground sprayers, UAV-based spraying has advantages in labor savings, operator health risk reduction, and flexibility: it is appropriate for hilly and mountainous areas where fruit trees are frequently cultivated and are on a steep slope or terrace with complex planting. There are several distinct types of UAVs that have been developed, each with a different number of rotors, payload capacity, and flying method [23]. In order to achieve control efficacy in fruit trees, certain models or operations have been developed for spraying fruit trees, whose 3D structure necessitates greater downwash and relatively large volumes of spraying. Recently launched UAV models with high payload capacities (more than 20 L) were tested for droplet deposition in apples, peaches, and pears [15,16,18,20,24]; however, comparisons to small models were seldom made. According to [16], the DJI Agras T30, a large model with a 30 L payload capacity, outperformed the Agras T20 with that of 20 L in spray coverage, particularly in the inner layer of citrus tree crowns, which led to greater droplet penetration of T30 irrespective of the inclusion of a tank-mix adjuvant. On the other hand, rotating flight, the ability to hover above each tree for spot spraying, was available in some models, such as the Agras series (Shenzhen DJI Innovation Technology) and P series (XAG). However, there had been a limited number of studies related to rotating flight, one of which examined droplet deposition in pear orchards [24]. To determine the viability of these approaches (i.e., large UAV models and rotating flight) for fruit trees in UAV applications, their effect on droplet deposition and control efficacy must be examined.

The majority of prior research on UAV-based spraying has focused on the features of spray droplet deposition in the canopy (distribution, droplet size, coverage, etc.) for comparison in spray circumstances (flight speed, volume, flight route, etc.) [25]. For example, a higher spray volume and flow rate and lower flight speed resulted in more droplet deposition in peaches and pears [15,18]. Increased flight altitude caused a significant decrease in drop deposition on the canopy; thus, an altitude of less than 2.5 m above the tree crown was recommended to reduce droplet drifting in a previous study [25]. Furthermore, due to the 3D structure of trees, tree shape has a significant impact on droplet deposition in citrus and peaches [15,26,27]. Although the premise behind this research was that droplet deposition and pest control efficacy were closely related, this assumption has only rarely been proven correct because of a lack of studies on pest control efficacy. In accordance with [27], tree shape had a substantial impact on droplet deposition, and manual spraying had 65–75% in terms of control efficacy in preventing citrus leafminer (*Phyllocnistis ciirella* Stainton) from infesting citrus trees. In almonds, two UAV-sprayed plots and one ground-sprayed plot were compared for damage rate due to navel orangeworm (*Amyelois transitella* Walker) [21], but no comparison was made for the non-sprayed plot, and the control efficacy was unclear.

In this study, we assessed the applicability of spraying chestnuts with ultra-low volumes of insecticide using UAVs. First, we evaluated the control efficacy of three insecticides applied to young chestnut plants using a UAV. The effectiveness of two of these insecticides was examined on adult trees. Additionally, we analyzed the spray coverage with regard to flying method, spray volume, type of UAV (i.e., payload capacity), flow rate, and tree age (i.e., tree shape) in order to characterize the droplet deposition of the spray solution.

## 2. Results

### 2.1. Control Efficacy of Ultra-Low-Volume Spraying Using UAV on Young Chestnut Trees

To test the control efficacy of UAV-based spraying, we used three pesticides, spinetoram and flubendiamide for peach moth and fluvalinate for chestnut weevil, in ultra-low volume because of their high concentration. We observed no side effects from the ultra-low-volume application of these pesticides on chestnut trees. The result is summarized in Table 1. Based on evaluation using 3–5 young trees of the cultivar ‘Porotan’ at GRIAT in 2020, the infestation rate of the not-spraying plot by peach moth was 6.8%, significantly higher than 1.1% of UAV-based spraying and 1.5% of conventional spraying with spinetoram (*p* = 0.005 and 0.013, respectively; Dunnett test); consequently, the control efficacy of UAV-based spraying was 84, equivalent to that of conventional spraying (78). In 2021, the efficacy using UAV was 67, slightly higher than conventional spraying with flubendiamide (53), although the infestation rate of these plots was not significantly different from not-spraying, which might be due to the relatively lower infestation rate and its variance among tree repetitions (4.1 ± 5.3 of the mean ± SD in the not-spraying plot). Additionally, when using flubendiamide (180 g ha^−1^), UAV-based spraying plots had a significantly lower infestation rate than not-spraying plots in 2020 (*p* = 0.008; Dunnett test), resulting in 92% control efficacy. In the other two years (2021 and 2022), the halved loading amount of flubendiamide (90 g ha^−1^) in two cultivars (‘Porotan’ and ‘Tsukuba’) was tested. The infestation rate of UAV and conventional spraying plots was significantly lower than that of not-spraying (*p* < 0.1; Dunnett test), except for 2021 in ‘Porotan’, the same as the case of spinetoram mentioned above.

For chestnut weevil, fluvalinate (200 g ha^−1^) was sprayed on young trees of mid-maturing cultivars ‘Riheiguri’ and ‘Mikuri’ because early maturing trees were not susceptible owing to the seasonal pattern of its emergence [28]. The infestation rate (%) of ‘Mikuri’ in the not-spraying plot in 2020–2022 was 52.5 ± 21.8, 1.5 ± 1.1, and 32.4 ± 20.3 of the mean ± SD, respectively (Table 2); thus, its large variances among years and tree repetition were evident, as reported in [29]. A significantly decreased infestation rate in the UAV-based plot (18.4 ± 29.8%) was observed in 2020 (*p* = 0.033; Welch’s *t*-test), in which the control efficacy was 65. A halved loading amount of fluvalinate (100 g ha^−1^) would not affect control efficacy (90 and 70 in 2021 and 2022, respectively), although statistical significance in the infestation rate with the not-spraying plot was not observed (Table 2; *p* > 0.1), which might be due to the large variance and the limited number of samples. Conventional spraying had no or less efficacy (53) than UAV-based spraying, which might be due to a reduced amount of fluvalinate. In ‘Riheiguri’, the UAV-based spraying had moderate control efficacy (58 and 83) in 2020 and 2021, respectively, with no significant difference to the not-spraying plot (*p* > 0.1; Welch’s *t*-test), while its high control efficacy (96) and significantly lower infestation rates were observed in 2022 (Table 2; *p* = 0.008; Welch’s *t*-test). Thus, pesticide spraying using UAVs would be effective in protecting young chestnut trees.

### 2.2. Protection of Adult Trees by UAV-Based Spraying of Pesticides Was Equivalent to Conventional Spraying

**Figure 1:**
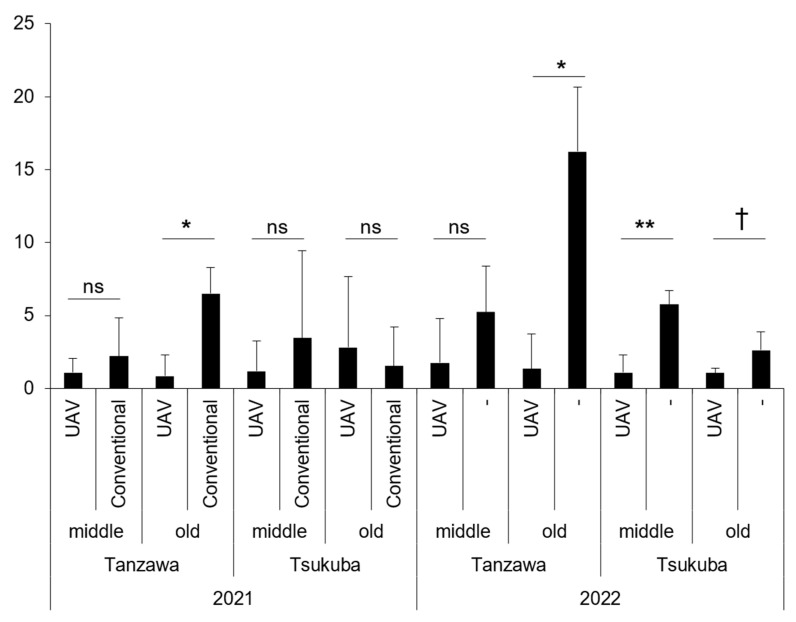
Infestation rate (%) by peach moth under control with flubendiamide on adult chestnut trees over two years (2021–2022). Error bars represent the standard deviation. The spraying methods used were as follows: UAV (UAV-based ultra-low-volume spraying), conventional (spraying with an air-blast sprayer), “-” (not sprayed). The categories “middle” and “old” refer to tree age, with “middle” indicating 8–15 years old and “old” indicating more than 15 years old. The statistical significance of the difference in mean values was determined using a two-tailed Welch’s *t*-test and is indicated as follows: ns (not significant at the 10% level), † *p* < 0.10, * *p* < 0.05, ** *p* < 0.01.

We used adult chestnut trees adhering to four criteria (two tree ages [middle age, 8–15 years old; old age, over 15 years old] and two cultivars [‘Tanzawa’ and ‘Tsukuba’]) to evaluate the applicability of aerial control using UAV in terms of adult trees. The spraying of flubendiamide using a UAV was as effective as conventional spraying (using an air-blast sprayer) in any criteria (i.e., tree ages and cultivars) based on the infestation rate by peach moth in 2021, of which the old trees of ‘Tanzawa’ of the UAV-based spraying plot (0.8% of the mean) were even significantly lower than those of conventional spraying (6.5%, Figure 1, Table S1) (*p* < 0.014; two-tailed Welch’s *t*-test). In 2022, the not-spraying plot had 2.6–16.2% (old ‘Tsukuba’ and old ‘Tanzawa’, respectively) in terms of infestation rate, while lower values were observed in the UAV-based spraying plot, with statistical significance in three out of the four criteria (*p* = 0.046, 0.004 and 0.083 for old ‘Tanzawa’, middle, and old ‘Tsukuba’, respectively; one-tailed Welch’s *t*-test), whose control efficacy was 59–92 (Figure 1, Table S1).

**Figure 2:**
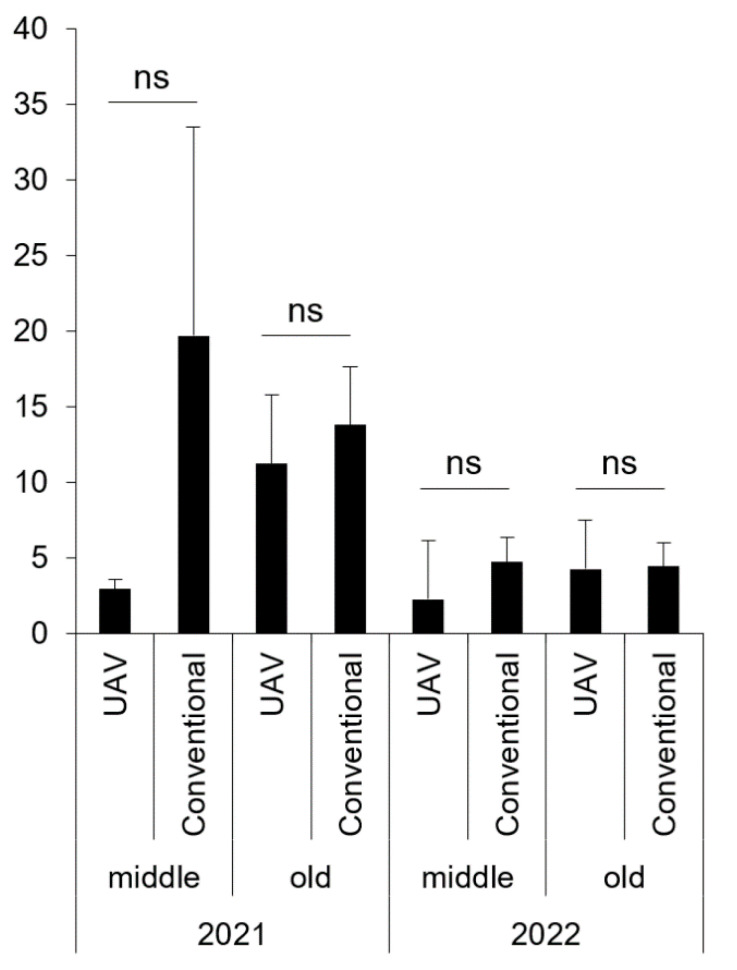
Infestation rate (%) by chestnut weevil under control with fluvalinate on adult ‘Tsukuba’ chestnut trees over two years (2021–2022). Error bars represent the standard deviation. The spraying methods used were as follows: UAV (UAV-based ultra-low-volume spraying) and conventional (spraying with an air-blast sprayer). The categories “middle” and “old” refer to tree age, with “middle” indicating 8–15 years old and “old” indicating more than 15 years old. “ns” indicates that the difference in mean values is not significant at the 10% level, as determined by a two-tailed Welch’s *t*-test.

Fluvalinate was sprayed using a UAV on adult trees of “Tsukuba” in both years for the control of chestnut weevil, whose infestation rate of the UAV-based spraying plot was lower than conventional spraying (other pesticides containing cypermethrin were used) in the mean value of any criteria although they showed no statistical significance (Figure 2, Table S2; *p* > 0.1; two-tailed Welch’s *t*-test). The largest difference in mean of the two plots was observed on trees at middle age in 2021 (2.9 ± 0.7% [UAV-based spraying] vs. 19.7 ± 13.8% [conventional] of mean ± SD). Overall, these results showed that the control efficacy of UAV-based spraying of peach moth and chestnut weevil could be equivalent to that of conventional spraying.

### 2.3. Droplet Deposition of UAV-Based Spraying onto Chestnut Trees

**Figure 3:**
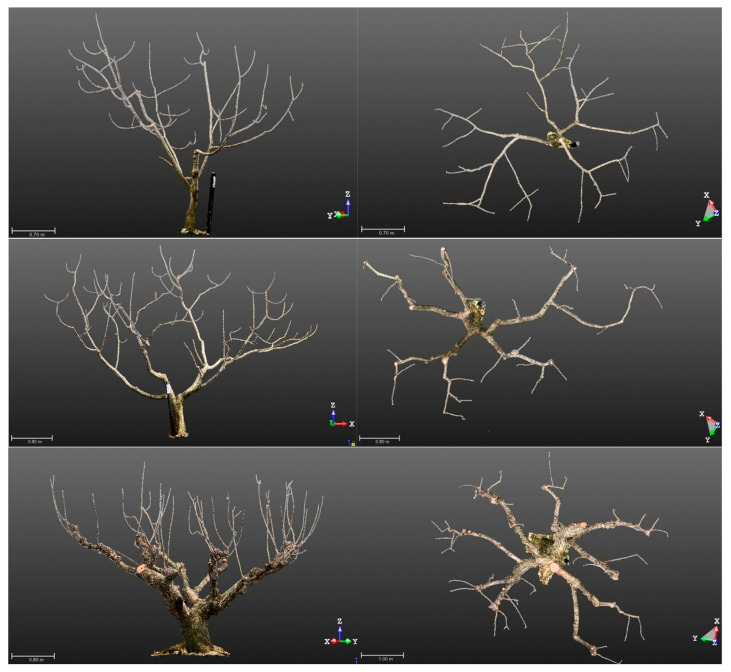
Snapshot of a dense 3D point cloud of representative chestnut trees after winter pruning. The **left** and **right** images represent the lateral and top views, respectively. The **upper**, **middle**, and **lower** images correspond to a young tree, an adult tree in middle age, and an adult tree in old age, respectively. Note that the upper part of the trunk of the young tree has been pruned to limit tree height, resulting in a modified central leader structure. The scale in meters is indicated by the bars at the lower left of each image, and the orientation in 3D space is indicated by the arrows at the lower right. A black pole near the tree trunk was used for 3D point cloud registration.

**Figure 4:**
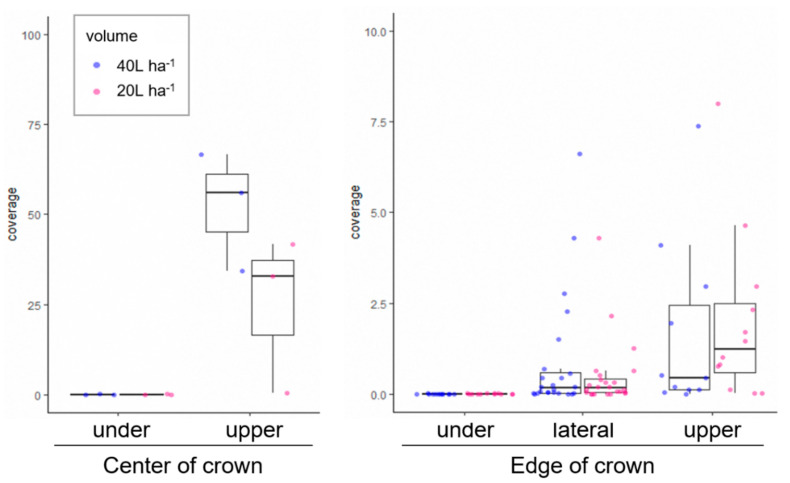
Distribution of spray coverage (%) by rotating flight for UAV-based spraying on young trees. Spraying volumes (20 L vs. 40L ha^−1^) were compared (trial no. 1 in Table 3). The heights of the position of WSP at the center and edge of the crown are 3.0 m and 1.5 m, respectively.

To characterize the droplet deposition of spraying with UAVs onto chestnut trees, we measured the spray coverage under different conditions as follows: spray volume, flight method (rotating flight for each tree and straight flight), the size of the UAV (i.e., payload capacity), flow rate, and tree age. For comparison of the former two, a four-rotor UAV, P20 (Table 4), sprayed water onto young trees with a central leader structure (Figure 3). Spraying 40 L ha^−1^ of spray volume with rotating flight, the median of spray coverage on the upper side at the center of the crown of 3.0 m height was 56.0%, which was higher than that of 20 L ha^−1^ (32.7%) (Figure 4). At the edge of the crown (1.5 m height), the median of spray coverage on the lateral side and upper side of 20 L ha^−1^ was about 0.2% and 1.2%, respectively, while no obvious differences were observed among spray volumes (Figure 4). The underside had nearly zero spray coverage at any location of the crown. These tendencies were observed when spraying pesticides in 2020 (Figure S1). Straight flight of spraying 20 L ha^−1^ was 7.8% of the median of spray coverage on the upper side at the center of the crown, 0.2% and 1.3% of that of the lateral side and upper side at the edge, respectively, when spraying pesticides in 2022 with T10 UAV (Figure S2), and the values of the upper side were significantly different among the locations of the crown (i.e., center vs. edge; *p* = 0.0011; Wilcoxon rank sum test). Note that these data were different from rotating flight under the experimental conditions (date, UAV, spraying agent, etc.).

**Figure 5:**
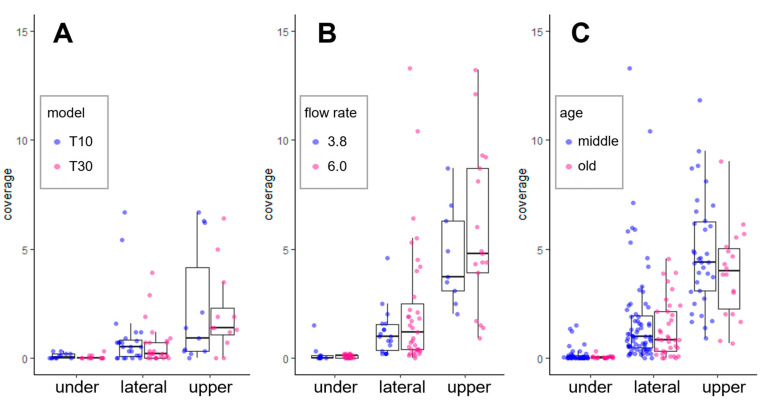
Distribution of spray coverage (%) of straight flight for UAV-based spraying on adult trees. The height of the position of WSP at the edge of the crown of an adult tree in middle and old age is 3.0 m and 3.5 m, respectively. A comparison was made for the followings: (**A**) the size of the UAVs (8 L and 30 L in payload capacity; trial no. 9 in Table 3), (**B**) the flow rate (3.8 L vs. 6.0 L min^−1^; trial no. 13), (**C**) the tree shape (middle vs. old; trial no. 11–13).

The size of the UAV (T10 and T30; payload capacities of 8 L and 30 L, respectively; Table 4) and flow rate were compared in adult trees in middle and old age, respectively, the pruning of which was to limit 4–5 m of tree height during summer and locate most fruit-bearing branches at the edge of the crown (Figure 3). Spraying water of 20 L ha^−1^ with T10, the median of spray coverage at the edge of the crown (3.0 m height) was 0.5% and 1.0% on the lateral side and upper side, respectively (Figure 5A), which showed no significant difference when spraying with T30. In 6.0 L min^−1^ of flow rate, the median value of spray coverage at the edge of the crown (3.5 m height) was 4.4%, slightly higher than that of 3.8 L min^−1^ (3.7%) (Figure 5B) when spraying flubendiamide of 40 L ha^−1^ in 2022 (trial no. 13 in Table 3).

The shape of the adult tree was different between middle and old age mainly due to length and the number of fruit-bearing branches: trees in middle age have relatively short (30–50 cm) and 4–6 branches per m^2^ of crown, while old trees have long (1–2 m) and 2–3 branches per m^2^ (Figure 3). Spraying the pesticides of 40 L ha^−1^ in 2021–2022 (trial no. 11–13), the median spray coverage of the upper side at the edge of the crown of the middle trees was 4.6%, which was slightly higher than that of the old (4.1%) (Figure 5C). This might be because of the height of the fruit’s location from ground level (3.0 m [middle] vs. 3.5 m [old]) (Figure 3).

## 3. Discussion

We demonstrate that UAV-based application of ultra-low-volume pesticides can be effective in protecting chestnut trees. Although no spray coverage on the underside, even using larger sizes of UAV (T20K and T30 in this study), raised concerns that the chemical without systemic or translaminar activity would not be effective, we confirmed that UAV-based pesticide application was equivalent to conventional spraying in terms of control efficacy irrespective of the models used, even if its active ingredient did not have such properties. This might be because the protection targets of pesticide (i.e., target of pest attack) are mainly chestnut fruits, which are located on the upper side of the branches and periphery of the tree crown; thus, UAV-based spraying from above the tree would be effective in maintaining certain droplet deposition on the fruits. In fact, two-thirds of the on-tree chestnut fruits were visible from directly above the tree [10]. These characteristics and pruning practice (discussed in the following) might contribute to the fact that the control efficacy was sufficient even at lower amounts than those applied to other tree species (20–40 L ha^−1^ [this study] vs. > 40 L ha^−1^ [almond, pear, citrus, etc.]) [16,18,21].

Tree shape would have a significant effect on the efficacy of pesticides. For example, in a straight flight, the degree of spray coverage at the edge of the crown (1.5 m height and 1.5 m width) was about 1/4 of that at the center of the crown (3.0 m high). This result and the tall height of chestnut trees without pruning [1,6] imply that if chestnut trees would have higher height and bore fruits at lower parts with the wider crown, the droplet deposition of UAV spraying might be scarce, resulting in little or no control effect on the fruit at the edge. The low-tree-height pruning method implemented in Gifu Prefecture keeps the height of adult trees below a certain level (4–5 m in summer) and at a constant density of fruit-bearing branches [30], which is likely to achieve better droplet deposition and control efficacy in terms of the application of pesticides onto the fruits, irrespective of tree age. In fact, the spray coverage was above a certain level (about 1% at 20 L ha^−1^ and more than 4% at 40 L ha^−1^) at the edge of adult trees, and there was no clear difference in the control efficacy of flubediamide against the peach moth between young and adult trees.

Rotating flight for each tree outperformed straight flight in spray coverage at the center of the crown of the young tree’s central leader structure, suggesting that this flight method would be effective in protecting the tree and that it is suitable for sparse planting despite the fact that more flight time is necessary. On the other hand, no significant effect in terms of flow rate, the size of UAVs, or the age of adult trees on spray coverage was observed, which might be because of the lower spray volume than previous reports (discussed above) and the limited number of measurement repetitions. Further study is necessary to determine optimal flight conditions, such as flight speed and nozzle type.

Interestingly, when the amount of fluvalinate was reduced by half (100 g ha^−1^), the control efficacy of the UAV-based spraying was higher than that of conventional spraying on young trees. This result suggests that a high concentration (100-fold the dilution of conventional spraying) might be more effective or more likely to sustain its efficacy in fluvalinate. Further validation of control efficacy on adult trees is needed. Moreover, this result suggests that UAV-based applications could realize a reduction in pesticide use, which is beneficial to the environment and health of related animals, as proposed in policies such as the Farm-to-Folk strategy in Europe and the Strategy for Sustainable Food Systems in Japan. Variable rate or site-specific UAV spraying would be possible by coupling with remote sensing of chestnut trees and their on-tree fruits [10,11,12,13] to reduce pesticide use, as proposed in weed and disease control [31,32].

## 4. Materials and Methods

### 4.1. Orchard Spray Events and Testing Layout

The study was conducted at the Gifu Prefectural Research Institute for Agricultural Technology (GRIAT) in Nakatsugawa, Gifu, Japan (35°29′ N, 137°28′ E) over a three-year period (2020–2022). The chestnut field covered an area of 0.4 hectares and consisted of grafting clones of Japanese chestnut (*Castanea crenata* Sieb. et Zucc.) cultivars, including ‘Tanzawa’, ‘Porotan’, and ‘Tsukuba’ as well as hybrids with Chinese chestnut (*C. mollissima* Blume) such as ‘Riheiguri’ and ‘Mikuri’. The trees were planted in rows with five trees per row, spaced at 5 m × 5 m (400 trees per hectare). The experimental plots within the field consisted of 2–5 trees in each row.

The 1.2-ha chestnut field at Sakashita in Nakatsugawa (35°56′ N, 137°53′ E; Figure S3) was another field. The field had a similar plant density to the GRIAT field, but the majority of the trees were adult ones (50 years old at most) in one of two phases (middle age, 8–15 years old, or old age, above 15 years old). Two blocks of chestnut trees made up the experimental plots in Sakashita (Figure S3). A 0.7 ha block was designated for UAV-based applications, while a 0.5-ha block was designated for conventional or unsprayed plots. Farmers in the Gifu Prefecture conducted pruning practices every winter at both fields in accordance with commercial best practices, which were developed to avoid alternate bearing, ensuring high-quality nuts (large nut weight and low rate of damaged nuts) and extending tree age [30].

Ten spray experiments for young trees at the GRIAT field and four for older trees at Sakashita field were carried out for control efficacy and spray coverage assessment in August (Table 3). The second-generation peach moth (*Conogethes punctiferalis* Guenée) laid eggs, and in August, those eggs began to hatch. The chestnut weevil (*Curculio sikkimensis* Heller), which targets mostly cultivars in the middle of maturation (i.e., the harvest season began in the middle of September in Nakatsugawa, Japan), emerged from underground in late August and early September [28]. These times correspond to the phases of fruit growth and maturation for the cultivars of chestnut used in this study—‘Tanzawa’, ‘Porotan’, ‘Riheiguri’, ‘Mikuri’, and ‘Tsukuba’—whose harvest dates began in early September for ‘Tanzawa’, middle September for ‘Porotan’, and late September for ‘Riheiguri’, ‘Mikuri’, and ‘Tsukuba’.

**Table 3:** Trials of ultra-low-volume applications using UAVs.

Trial No.	Location	Year	Date	Chemical Agent ^a^	Volume (L ha^−1^)	UAVs	Flight Mode	Operation	Speed (m s^−1^)	Spray Coverage	Weather	Temp.^f^ (°C)	RH ^f^ (%)	Wind ^g^ (m s^−1^)
1	GRIAT	2020	31 July	NA (water) ^b^	20, 40	P20	Rotating	Auto	2.0	+	Sunny	28.7	59.0	W, 2–3
2			4 August	Spinetoram	20				2.0	+	Sunny	23.7	86.0	NW, 0–1
3			13 August	Flubendiamide	40				2.0	+	Cloudy	25.9	79.8	E, 1–2
4			24 August	Fluvalinate	40				2.0	+	Sunny	23.3	78.0	NW, 1–2
5		2021	4 August	Spinetoram	20	TDS-AG10	Straight	Manual	3.5	-	Sunny	25.3	76.9	ND
6			27 August	Flubendiamide	20				2.6	-	Sunny	24.6	80.5	E, 1–2
7				Fluvalinate	20				2.9	-				
8		2022	19 August	Flubendiamide	20	T10	Straight	Manual	3.4	+	Sunny	28.6	46.8	SW, 0–1
9				NA (water)	20	T10, T30		Auto	2.8	+		27.2	58.4	SW, 0–1
10			26 August	Fluvalinate	20	T10		Manual	3.7	+	Sunny	23.4	83.0	ND
11	Sakashita	2021	4 August	Flubendiamide	40 ^d^	T20K	Straight	Auto	4.3	+	Sunny	31.7	49.6	ND
12			11 August	Fluvalinate ^c^	40 ^d^				4.3	+	Cloudy	24.4	76.1	ND
13		2022	1 August	Flubendiamide	40	T20K	Straight	Auto	4.1/5.8 ^e^	+	Sunny	32.7	54.3	S, 0–2
14			16 August	Fluvalinate ^c^	40				4.1/5.8	-	Cloudy	27.7	78.9	ND

The abbreviations were as follows: Temp., temperature; RH, relative humidity. ^a^ The concentrations of the active ingredients (ppm) in conventional UAV-based spraying of spinetoram, flubendiamide, and fluvalinate are 2500 (25), 4500 (45), and 5000 (100), respectively. ^b^ NA, not applicable; water was sprayed for spray coverage evaluation. ^c^ For the conventional spraying with a volume of 2000 L ha^−1^, a different chemical agent, cypermethrin at 20 ppm, was used. ^d^ Interval of a straight flight route is 2.5 m, while that for others is 5.0 m. ^e^ Application speed is changed according to flow rate (3.8 L min^−1^ for 4.1 m s^−1^ and 6.0 L min^−1^ for 5.8 m s^−1)^. ^f^ Data were obtained from the Agro-Meteorological Grid Square Data of the National Agriculture and Food Research Organization [33]. ^g^ Wind condition is indicated as direction and speed. ND, not detected.

### 4.2. UAV and Spray System

**Figure 6:**
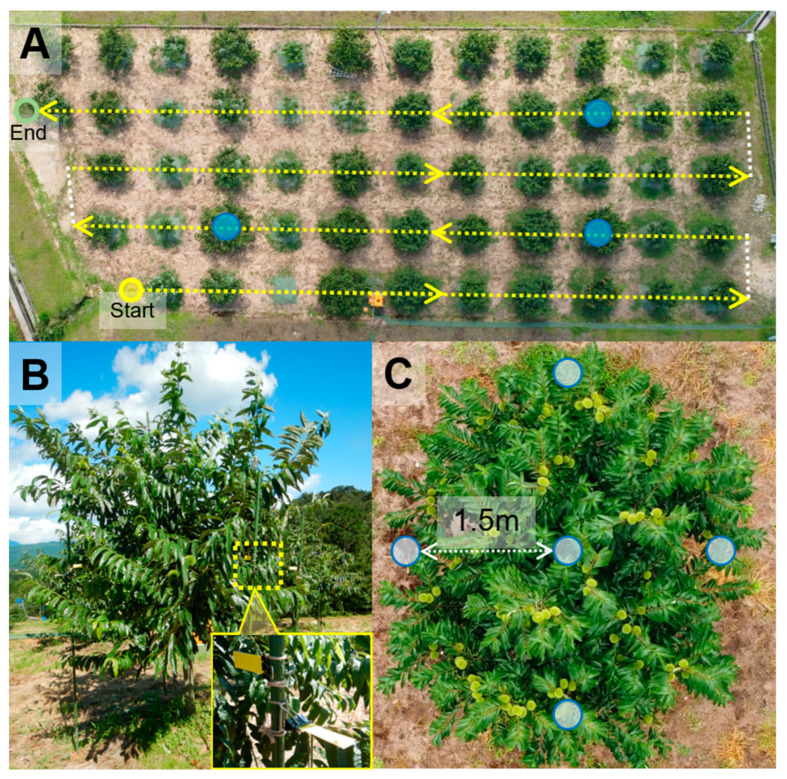
Layout of the straight flight by unmanned aerial vehicle (UAV) (**A**), side view (**B**), and top view (**C**) of a young chestnut tree for spray coverage evaluation. The yellow dotted arrows represent the route of the UAV during spraying, while the white dotted lines indicate the route without spraying (trial no. 9 in Table 3). The blue circles indicate the trees where water-sensitive paper (WSP) was placed. In a lower left image, a side view of a representative tree showing vertically and horizontally placed WSPs using poles and clips as representative location of the on-tree fruits at the edge and center of the crown, positioned at a height of 1.5 m and 3.0 m, respectively. The light blue circles in the lower right image indicate the locations where the papers were attached.

The UAVs deployed included the P20 (XAG, Guangzhou, China), TDS-AG10 (TDS, Kasugai, Japan), T20K (Kubota, Osaka, Japan; the product was comparable to the Agras T20 from DJI), Agras T10 (Shenzhen DJI Innovation Technology, Shenzhen, China), and T30 (Shenzhen DJI Innovation Technology). Table 4 includes a list of the aerial platform’s specifications and spray characteristics. T20K and T30 have larger payload capacities (16 L and 30 L, respectively) than the other models, which is also the case for the spray width and nozzle number. There were two types of nozzles: a rotary disc centrifugal nozzle for P20 and a flat-fan hydraulic nozzle for the others [25]. The spraying volume for the UAV-based pesticide application was set at 20–40 L ha^−1^ (Table 3). UAVs were flown centered above the trees for each row with multiple round trips in parallel to the row orientation through manual operation at the GRIAT field (trial no. 5–8 and 10 in Table 3), while GPS-guided autonomous flight with 2.5 and 5.0 m intervals between rows was carried out at the Sakashita field in 2021 and 2022, respectively (trial no. 11–14). At GRIAT, rotating flight (trial no. 1–4) and water spraying (trial no. 1 and 9) were carried out autonomously for the purpose of evaluating spray coverage and comparing spray volume and the UAV model (Figure 6). The spraying height was above 2–3 m from the tree’s crown (about 6–7 m above ground). At the fields of GRIAT and Sakashita, conventional spraying was performed using high-pressure sprayers (SHPE2025DX; Kioritz, Tokyo, Japan) and air-blast sprayers (SSV553F; Kioritz, Tokyo, Japan), respectively.

**Table 4:** Summary of the species of UAVs used in this study.

	Spray Width (m)	Nozzle		Flow Rate (L min^−1^)	Droplet Size (μm)	Payload Capacity (L)
Model		Model ^b^	No.			
P20	5	SNZ-14000A	4	2.4	90–300	10
TDS-AG10	3	VP110015	2	0.8	136–177	10
Agras T10	5.5	XR11001VS	4	1.7	130–250	8
T20K ^a^	7	XR11001VS ^c^	8	2.5	130–250	16
		XR110015VS		3.8, 6.0	170–265	
Agras T30	9	XR11001VS	16	1.7	130–250	30

^a^ The T20K from Kubota is an equivalent product to the Agras T20 from Shenzhen DJI Innovation Technology. ^b^ Manufacturer of nozzles were the followings: XAG for SNZ-14000A, Hypro (New Brighton, MN, USA) for VP110015 and TeeJet Technologies (Glendale Heights, IL, USA) for XR11001VS/XR110015VS. ^c^ XR11001VS was used for the test in 2021 and XR110015VS in 2022.

### 4.3. Pesticides Used in This Study

In this investigation, peach moth was controlled using DIANA^®^ WDG (Water Dispersible Granule; Sumitomo Chemical, Tokyo, Japan), which includes 25% spinetoram as an active ingredient (a.i.). Because of its effects on the nicotinic acetylcholine receptor and the gamma-aminobutyric acid receptor, spinetoram is categorized as a group 5 pesticide in a pesticide mode of action (MoA), as published by the Pesticide Resistance Action Committee [34]. It also has translaminar activity and a broad insecticidal spectrum. Spinetoram, 50 g a.i. ha^−1^ was used in this investigation. The active component of PHOENIX (Nihon Nohyaku, Tokyo, Japan), a flowable type of pesticide, is 18% flubendiamide, which is also used to control peach moths. Due to its effects on sodium channels, flubendiamide is categorized as group 3A in MoA and has a highly selective activity against pest Lepidoptera species [35]. In this trial, flubendiamide was utilized at doses of 90 or 180 g a.i. ha^−1^. 20% *tau*-fluvalinate is the active component of MAVRIK-WP (Nihon Nohyaku), a water-soluble powder that is used to suppress chestnut weevils. *Tau*-fluvalinate, which is categorized as group 3A in MoA, has a wide insecticidal spectrum (lepidoptera, thysanoptera, and aphids, among others), an antifeeding action, and a repelling effect. In this investigation, tau-fluvalinate at 100 or 200 g a.i. ha^−1^ was utilized. The formulation type of emulsifiable concentrate Agrothrin^®^ (Sumitomo Chemical) comprises 6% cypermethrin as the active component, which is also effective against chestnut weevil. Cypermethrin is categorized as group 3A in MoA and has a wide insecticidal range. The amount of cypermethrin employed in this investigation was 40 g a.i. ha^−1^. In this investigation, no surfactant for reducing surface tension or spreading droplets was used.

### 4.4. Evaluation of Infestation Rate by Insect Pests and Spray Droplet Deposition

Over a three-year period (2020–2022), the infestation rates of peach moth and chestnut weevil were evaluated. For each tree, the nuts were collected, and those that had been harmed by infestation were chosen between early September and October. In GRIAT, the total number of nuts collected for peach moth and chestnut weevil infestation assessment reached 168 and 249 of the mean and 20 and 46 of the minimum, respectively. In Sakashita field, the total number of nuts harvested reached 66 and 83 of the mean and 29 and 31 of the minimum, respectively. The ratio of insect-damaged nuts to all the nuts gathered was used to calculate the infestation rate. The following is how control efficacy (%) was calculated: the ratio of the infestation rate of spraying plots to those without spraying plots was subtracted from 1.

Water-sensitive paper (WSP, 76 mm × 26 mm, Syngenta Crop Protection AG, Basel, Switzerland) was used to measure the spray coverage. We set WSPs using poles and clips to ensure reproducibility of the evaluation (Figure 6), not directly on leaves, as in previous reports, e.g., [18,20]. This is because the protection targets of the tested pesticides were mainly chestnut fruits, whose location in the canopy is thought to be highly variable among trees. A pair of these papers, one on the front and one on the back, were placed horizontally and vertically at the 1.5 m crown edge and only horizontally at the 3.0 m crown center in young trees as representative locations of the on-tree fruits (Figure 6). At the edge of the crown of adult trees, 3.0 and 3.5 m high for the trees in middle and old age, respectively, the WSP was positioned vertically and horizontally in three directions (Figure S4). Following the spraying, the papers were gathered and scanned using an MG6160 (Canon, Tokyo, Japan) scanner at 600 dpi, with the data being saved in TIFF format. Pictures of WSP that had been scanned were converted to 8-bit grayscale, and after that, the image analyzer program ImageJ^TM^ [36] was used to create binary pictures within a predetermined threshold, measure the masked regions, and calculate spray coverage for each image.

### 4.5. Data Analysis

R software version 4.3.0 [37] was used to conduct all statistical analyses. Using the “tidyverse” packages, a boxplot with jitter plots of the spray coverage data was created. Welch’s *t*-test and Dunnett test, the latter of which made use of the “multcomp” package, were used to infer the difference in infestation rate. For all analyses, statistical significance was inferred at *p* < 0.10. Due to the large within-plot variation, there was no statistically significant difference in spray coverage according to the Wilcoxon rank sum test if it was not stated in the text.

### 4.6. Terrestrial Laser Scanner

Light detection and ranging technology were used to visualize tree-training practices at each phase (i.e., age). In early December 2021, at the Sakashita field, we employed a laser scanner, the FARO^®^ FocusS150 (FARO Technologies, Lake Mary, FL, USA), to gather the point cloud data of the structure of chestnut trees after pruning. With a setting of 1/4 scan resolution and 4 scan quality (a maximum measurement rate of 976,000 points per second), it scans with a visual field of 300° vertically and 360° horizontally from 12 positions around a tree for young age or 16 positions for old age, resulting in an average 3D position accuracy of 7.7 mm at 10 m. Trimble^®^ RealWorks Viewer (Trimble Geospatial, Westminster, CO, USA) was used to display the 3D point cloud.

## 5. Conclusions

Our results suggest that UAV-based pesticide application is promising for the control of peach moth and chestnut weevil. Its control efficacy was robust in terms of spray conditions (flight method, UAV model, and spray volume) and tree age. No significant effects of spray volume, flight method, size of UAVs, flow rate, or age of the adult tree on spray coverage of UAV-based spraying were observed under our experimental conditions, whereas the difference in spray coverage among the location of the crown (i.e., center vs. edge) of the central leader structure suggested the importance of pruning and tree height to ensure control efficacy. Spraying pesticides using a UAV is beneficial to farmers in terms of both saving labor and reducing the risk to their health. The optimization of spray conditions and the possibility of reducing pesticide use should be investigated in the future.

## Figures and Tables

**Table 1:** Summary of the efficacy of the control of infestations by peach moths on young trees.

Cultivar	Year	Pesticide	Spraying	Volume	*n*	Infestation Rate (%)	Efficacy	*p*-Value
Porotan	2020	-	-	-	5	6.8 ± 3.6	-	-
		spinetoram	UAV	20 L	5	1.1 ± 1.5	84	**0.005**
			Conventional	2000 L	4	1.5 ± 1.7	78	**0.013**
		flubendiamide	UAV	40 L	3	0.5 ± 0.9	92	**0.008**
	2021	-	-	-	3	4.1 ± 5.3	-	-
		spinetoram	UAV	20 L	5	1.4 ± 1.2	67	0.400
		flubendiamide	UAV	20 L	3	1.6 ± 1.9	61	0.547
			Conventional	2000 L	4	1.9 ± 1.8	53	0.590
	2022	-	-	-	3	5.3 ± 1.6	-	-
		flubendiamide	UAV	20 L	3	1.9 ± 0.7	65	**0.024**
			Conventional	2000 L	5	2.5 ± 1.5	53	**0.036**
Tsukuba	2021	-	-	-	3	3.7 ± 1.3	-	-
		flubendiamide	UAV	20 L	3	1.7 ± 1.3	53	**0.086**
			Conventional	2000 L	4	0.6 ± 0.3	84	**0.013**
	2022	-	-	-	4	8.6 ± 4.0	-	-
		flubendiamide	UAV	20 L	3	3.0 ± 1.5	65	**0.079**
			Conventional	2000 L	3	2.6 ± 2.2	70	**0.060**

The values of the infestation rate were indicated as the mean ± standard deviation. Spraying methods were the as follows: UAV, UAV-based ultra-low-volume spraying; conventional, spraying with a high-pressure sprayer; “-,” not sprayed. *p*-values were calculated using the Dunnett test (vs. not sprayed).

**Table 2:** Summary of control efficacy of infestation by chestnut weevil on young trees.

Cultivar	Year	Spraying	Volume	*n*	Infestation Rate (%)	Efficacy	*p*-Value
Riheiguri	2020	-	-	5	26.2 ± 10.2	-	-
		UAV	40 L	2	11.1 ± 12.4	58	0.125
	2021	-	-	5	2.2 ± 3.9	-	-
		UAV	20 L	2	0.4 ± 0.5	83	0.182
	2022	-	-	5	7.4 ± 4.9	-	-
		UAV	20 L	2	0.3 ± 0.4	96	**0.008**
Mikuri	2020	-	-	5	52.5 ± 21.8	-	-
		UAV	40 L	3	18.4 ± 29.8	65	**0.033**
	2021	-	-	3	1.5 ± 1.1	-	-
		UAV	20 L	4	0.1 ± 0.3	90	0.162
		Conventional	2000 L	2	1.9 ± 2.0	0	0.847
	2022	-	-	2	32.4 ± 20.3	-	-
		UAV	20 L	4	9.7 ± 8.7	70	0.114
		Conventional	2000 L	2	15.1 ± 3.1	53	0.295

The values of infestation rate were indicated as mean ± standard deviation. Spraying methods were as follows: UAV, UAV-based ultra-low-volume spraying; Conventional, spraying with high-pressure sprayer; “-,” not sprayed. *p*-values were calculated using Dunnett test (“Mikuri” in 2021 and 2022; vs. not sprayed) or one-tailed Welch’s *t*-test (others).

## Data Availability

Not applicable.

## Supplementary Materials

The following supporting information can be downloaded at: https://www.mdpi.com/article/10.3390/plants12142597/s1, Figure S1. Distribution of spray coverage (%) of rotating flights of UAV-based pesticide application on young trees. Figure S2. Distribution of spray coverage (%) of a straight flight of UAV-based pesticide application on young trees. Figure S3. A satellite image of the field at Sakashita. Figure S4. Layout of the straight flight by unmanned aerial vehicle (UAV) in trial no. 11–14 (A), adult chestnut tree viewed from obliquely (B) and vertically above (C) for evaluation of control efficacy of pesticides and spray coverage. Table S1. Infestation rate by peach moth under control with flubendiamide on adult chestnut trees over two years (2021–2022). Table S2. Infestation rate by chestnut weevil under control with fluvalinate on adult trees of chestnut cultivar ‘Tsukuba’ over two years (2021–2022).
