# Supplementary material for: Control Efficacy of UAV-Based Ultra-Low-Volume Application of Pesticide in Chestnut Orchards
Source: Plants (Basel). 2023 Jul 9;12(14):2597. doi: 10.3390/plants12142597 (PMC10384239; doi:10.3390/plants12142597)
Supplement: Supplementary file 1 [file plants-12-02597-s001.zip › Supplementary_Figures.pdf]

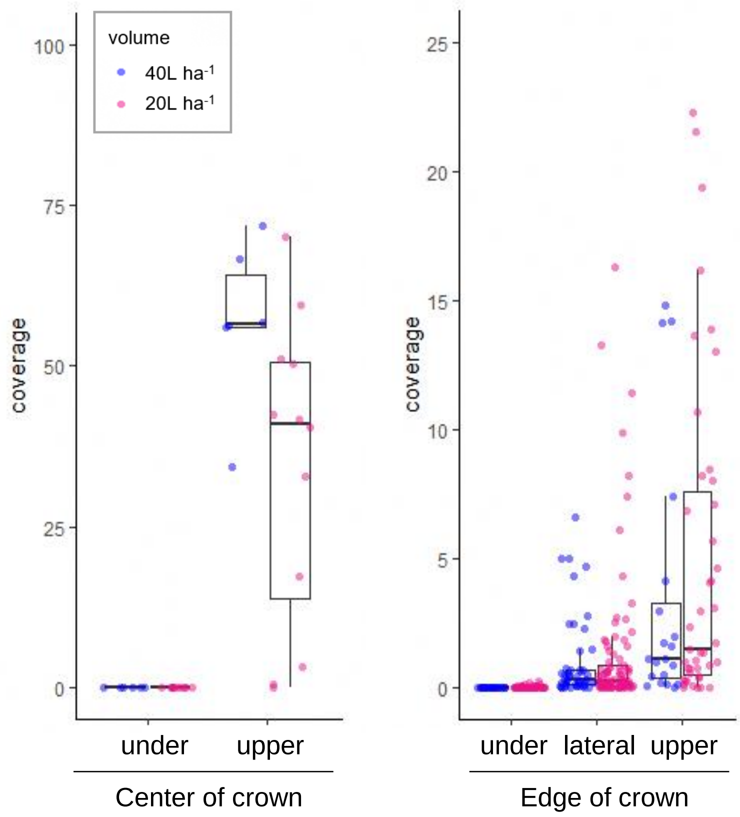

Figure S1. Distribution of spray coverage (%) of rotating flights of UAV-based pesticide application on young trees

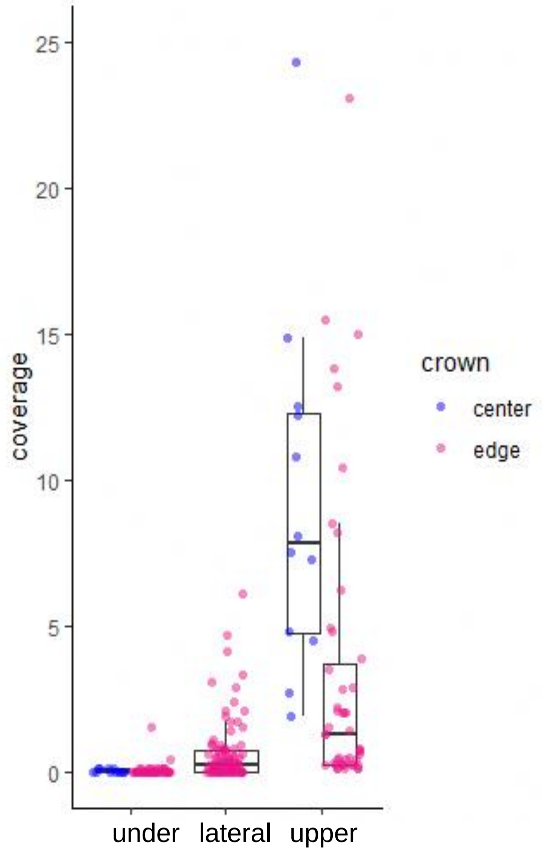

Figure S2. Distribution of spray coverage (%) of straight flights of UAV-based pesticide application on young trees

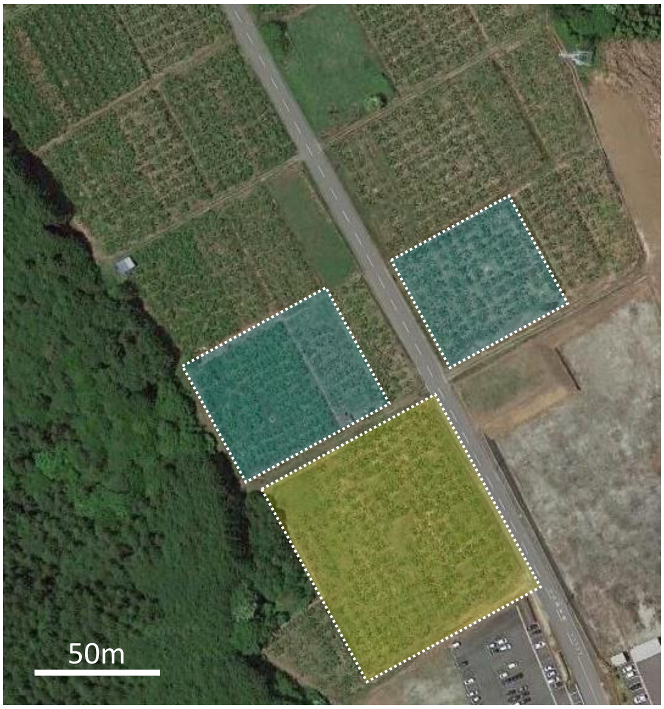

Figure S3. A satellite image of the field at Sakashita
This image, which points North on top, identified on the Google map. Yellow and light blue region surrounded by white dotted lines indicates the field for aerial and conventional spraying of pesticides, respectively, except for evaluation of peach moth infestation in 2022 (light blue region indicates not-sprayed plot).

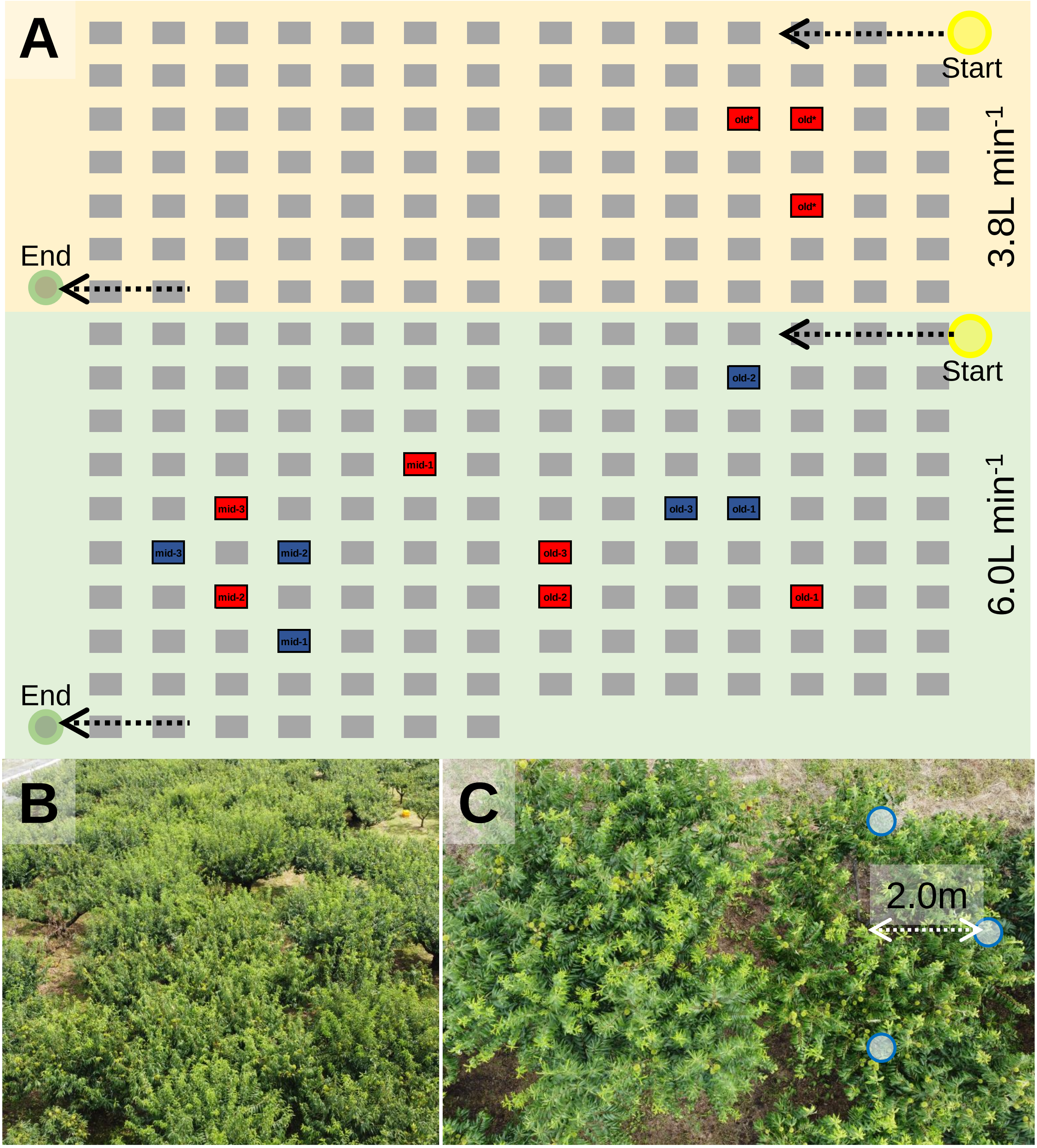

Figure S4. Layout of the straight flight by unmanned aerial vehicle (UAV) in trial no. 11–14 (A), adult chestnut tree viewed from obliquely (B) and vertically above (C) for evaluation of control efficacy of pesticides and spray coverage.

(A) Values at right (3.8L and 6.0L min^{-1}) indicate the flow rate of UAV-based spraying. Black dotted arrows indicate a route at start or end and a direction of straight fright with UAV. Gray boxes indicate the location of trees. Blue and red ones indicate the trees of cultivars ‘Tanzawa’ and ‘Tsukuba’, which are used for evaluation of the infestation rate by insects and spray coverage assessed by water sensitive paper. In these boxes, the age of adults trees (old and middle) is indicated as “old” and “mid”, respectively, whose attached number and asterisk with hyphen indicate the tree repetition for evaluation of both infestation rate and spray coverage and only the latter, respectively. (C) Light blue circles indicate the location of the water sensitive paper attached.
