# Supplementary material for: Control Efficacy of UAV-Based Ultra-Low-Volume Application of Pesticide in Chestnut Orchards
Source: Plants (Basel). 2023 Jul 9;12(14):2597. doi: 10.3390/plants12142597 (PMC10384239; doi:10.3390/plants12142597)
Supplement: Supplementary file 1 [file plants-12-02597-s001.zip › Supplementary_Tables.pdf]

Table S1. Infestation rate by peach moth under control with flubendiamide on adult chestnut trees over two years (2021–2022).

Year	Cultivar	Tree age	Spraying	Volume	Infestation rate (%)	Efficacy	p-value
2021	Tanzawa	middle	UAV	40L	1.1±1.0	-	0.541
			Conventional	2,000L	2.2±2.6	-	-
		old	UAV	40L	0.8±1.5	-	**0.014**
			Conventional	2,000L	6.5±1.8	-	-
	Tsukuba	middle	UAV	40L	1.2±2.1	-	0.588
			Conventional	2,000L	3.4±6.0	-	-
		old	UAV	40L	2.8±4.9	-	0.717
			Conventional	2,000L	1.5±2.7	-	-
2022	Tanzawa	middle	UAV	40L	1.8±3.0	66	0.103
			-	-	5.2±3.1	-	-
		old	UAV	40L	1.4±2.4	92	**0.046**
			-	-	16.2±4.4	-	-
	Tsukuba	middle	UAV	40L	1.1±1.2	81	**0.004**
			-	-	5.8±0.9	-	-
		old	UAV	40L	1.1±0.3	59	**0.083**
			-	-	2.6±1.3	-	-

The values of infestation rate were indicated as mean ± standard deviation.

Spraying methods were as following: UAV, UAV-based ultra-low-volume spraying; Conventional, spraying with air-blast sprayer; "-", not sprayed

P -values were calculated by Welch's *t* -test (two-tailed for comparison of UAV vs. Conventional; one-tailed for UAV vs. not sprayed)

Table S2. Infestation rate by chestnut weevil under control with fluvalinate on adult trees of chestnut cultivar ‘Tsukuba’ over two years (2021–2022).

Year	Tree age	Spraying	Volume	Infestation rate (%)	p-value
2021	middle	UAV	40L	2.9±0.7	0.174
		Conventional	2,000L	19.7±13.8	-
	old	UAV	40L	11.2±4.6	0.502
		Conventional	2,000L	13.8±3.9	-
2022	middle	UAV	40L	2.3±3.9	0.196
		Conventional	2,000L	4.7±1.6	-
	old	UAV	40L	4.3±3.3	0.468
		Conventional	2,000L	4.4±1.6	-

The values of infestation rate were indicated as mean ± standard deviation.

Spraying methods were as following: UAV, UAV-based ultra-low-volume spraying; Conventional, spraying with air-blast sprayer

P -values were calculated by two-tailed Welch's *t* -test
